# Nickel Content in Human Internal Organs

**DOI:** 10.1007/s12011-020-02347-w

**Published:** 2020-08-24

**Authors:** Danuta Dudek-Adamska, Teresa Lech, Tomasz Konopka, Paweł Kościelniak

**Affiliations:** 1 Departament of Analytical Chemistry, Faculty of Chemistry, Jagiellonian University in Kraków, Gronostajowa 2, 30-387 Kraków, Poland; 2 Institute of Forensic Research, Westerplatte 9, 31-033 Kraków, Poland; 3 Department of Forensic Medicine, Jagiellonian University Medical College, Grzegórzecka 6, 31-531 Kraków, Poland

**Keywords:** Nickel content, Human organs, Post-mortem material, ETAAS

## Abstract

With the growing interest in new applications of metals in modern technologies, an increase in their concentration in the environment can be observed, which, in consequence, may constitute a hazard to human health. That is why it is of a great importance to establish “reference” levels of particular elements (essential or toxic) in human biological samples.

The aim of this paper was to determine nickel in autopsy tissues of non-occupationally exposed subjects in Southern Poland (*n* = 60). Measurements were performed by means of electrothermal atomic absorption spectrometry after microwave-assisted acid digestion according to previously optimized and validated procedure. The results obtained indicate that data cover the wide range of concentrations and generally are consistent with other published findings. Nickel levels in the brain, stomach, liver, kidneys, lungs and heart (wet weight) were between 2.15–79.4 ng/g, 0.5–44.2 ng/g,7.85–519 ng/g, 12.8–725 ng/g, 8.47–333 ng/g and 2.3–97.7 ng/g, respectively. Females had generally lower levels of nickel in tissues than males (statistically significant relationships were found for the liver, kidneys and lungs), and median nickel concentrations in all studied material within all age groups had very similar values, with the exception of stomach.

## Introduction

Pollution by nickel (Ni) is ubiquitous. This is due to its natural abundance in the Earth’s crust (the 24th most naturally abundant element) and human activity [1, 2].

Nickel has an ever increasing number of applications in modern technologies. Its compounds are released into the environment at all stages of production and utilization and may constitute a hazard to human health [2, 3].

It has been proven that contact with nickel compounds (both soluble and insoluble) can lead to many pathological effects [2, 4], among which allergy in the form of contact dermatitis (estimated in 10–20% of the general population) is the most common and known reaction of the human body to nickel [2, 5, 6]. In addition, pulmonary fibrosis, respiratory tract cancer, iatrogenic intoxication, liver damage and cardiovascular and kidney diseases [1, 2, 7] have also been observed and may be considered as the consequence of selective deposition of nickel in the lungs, heart, diaphragm, brain and spinal cord following inhalation [7] and in bones, parenchymal organs, myocardium, skin, hair and various glands after ingestion [8]. When compared with controls, elevated levels in blood (mean ± SD: 3.0 ± 2.8 μg/L in controls and 25.5 ± 4.9 μg/L in iron and steel foundry workers [9]), urine (mean 4.36 vs 36.6 μg/L in steel production workers [10]) and body tissues (e.g. in the lungs of nickel refinery workers, mean 62 vs 24, 84 ng/g [11]) have been shown in occupationally exposed subjects. Though it is expected that exposure to nickel can occur from cigarette smoking, nickel concentration measured in smokers’ blood does not differ much from that determined in non-smokers’ blood [4].

Natural deficiencies of nickel practically do not occur, as typical daily intake with food (from 25 to 300 μg/day [2, 4, 7]) is more than three times the daily requirement [7]. In children, the average daily nickel intake was determined by age: 9, 38, 82 and 99 μg/day at 0–6 months, 7–12 months, 1–3 years and 4–8 years, respectively. In children aged 9–18 years, this value is similar to the average established for adults: 128–137 μg/day for men and 101–109 μg/day for women. Nickel-rich foods include porridge, dried beans and peas, nuts, dark chocolate and soy products [2]. Reference concentrations of nickel reported in the literature published at the end of the twentieth century vary from below 1.0 μg/kg in hair to 15.9 μg/kg in the lungs [12], and the established body burden of nickel in healthy and non-exposed adults averages about 7.3 μg/kg body weight [6].

Although data on the concentration of nickel in blood [13–18], urine [13–16, 19–22] or hair [14–16] can be found among recently published literature, only a few publications regarding nickel content in human internal organs are available. One of the newer papers, written in 2011 by Drobyshev and Aladyshkina [23], presents values for nickel only in the kidneys and liver for a very small population (*n* = 14). That is why in this study, an evaluation of nickel content in the internal organs of non-exposed and non-poisoned subjects from Southern Poland was carried out.

## Material and Methods

### Reagents and Instrumentation

All the reagents used (65% HNO_3_, 30% H_2_O_2_ and 1 g/L nickel stock solution) were supplied by Merck (Darmstadt, Germany) and were of analytical grade. Deionized water was obtained from NANOpure Diamond Water Purification System (Barnstead, Dubuque, IA).

Elemental analysis was carried out by an electrothermal atomic absorption spectrometer (Solaar MQZe, Thermo Electron, Waltham, MA), with Zeeman background correction, at a wavelength of 232.0 nm (slit width of 0.2 nm), according to a previously optimized and validated four-step procedure [24] after microwave-assisted acid digestion in an Ethos 1 microwave digestion system (Milestone, Sorisole, Italy) [25].

### Material and Sample Preparation

For evaluation of the reference ranges, sections of internal organs (brain, stomach, liver, kidneys, lungs and heart―weighing about 50–100 g) were taken from fresh 60 cadavers (within 24 h of death) during routinely performed autopsies at the Department of Forensic Medicine of the Jagiellonian University Medical College in Kraków (ethical clearance (KBET/102/B/2009) from the Bioethics Committee of the Jagiellonian University) in the years 2009–2010. The deceased subjects were from Southern Poland (21 women aged 29–89 years (mean 56 ± 18) and 39 men aged 24–88 years (mean 47 ± 13) as far as could be established were not environmentally or occupationally exposed to elevated levels of nickel nor intoxicated with any poisons and, on the basis of visual assessment of internal organs by a forensic medical doctor, were without any visible pathological or injury-related changes).

The following regions of internal organs were always sampled: cerebral cortex, superior surface of right lobe of the liver, kidney cortex and medulla, anterior surface of the lower lobe of the lung, body wall of the stomach and left ventricular muscle of the heart. Samples were deposited directly into the polypropylene collection vessels which had been previously soaked for 24 h in 5% (v/v) nitric acid solution and rinsed with deionized water and then kept frozen at − 20 °C until analysis in 2012/2013. Prior to determination, samples were thawed, homogenized and wet-digested in a mixture of nitric acid and hydrogen peroxide (v/v 5:1) [26].

### Statistical Analysis

**Fig. 1:**
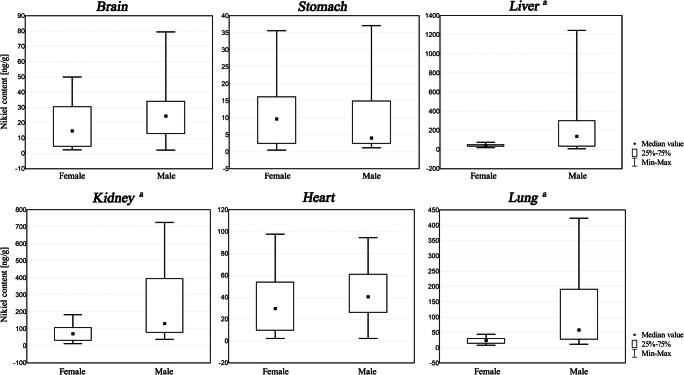
Nickel content in internal organs (ng/g) of the non-exposed population of Southern Poland: Mann-Whitney *U* test results presented in box and whisker plots according to gender. **a** Statistically significant relationships between nickel content and gender (*p* < 0.05) were revealed in the liver (*p* = 0.01), kidneys (*p* = 0.0006) and lungs (*p* = 0.001)

**Fig. 2:**
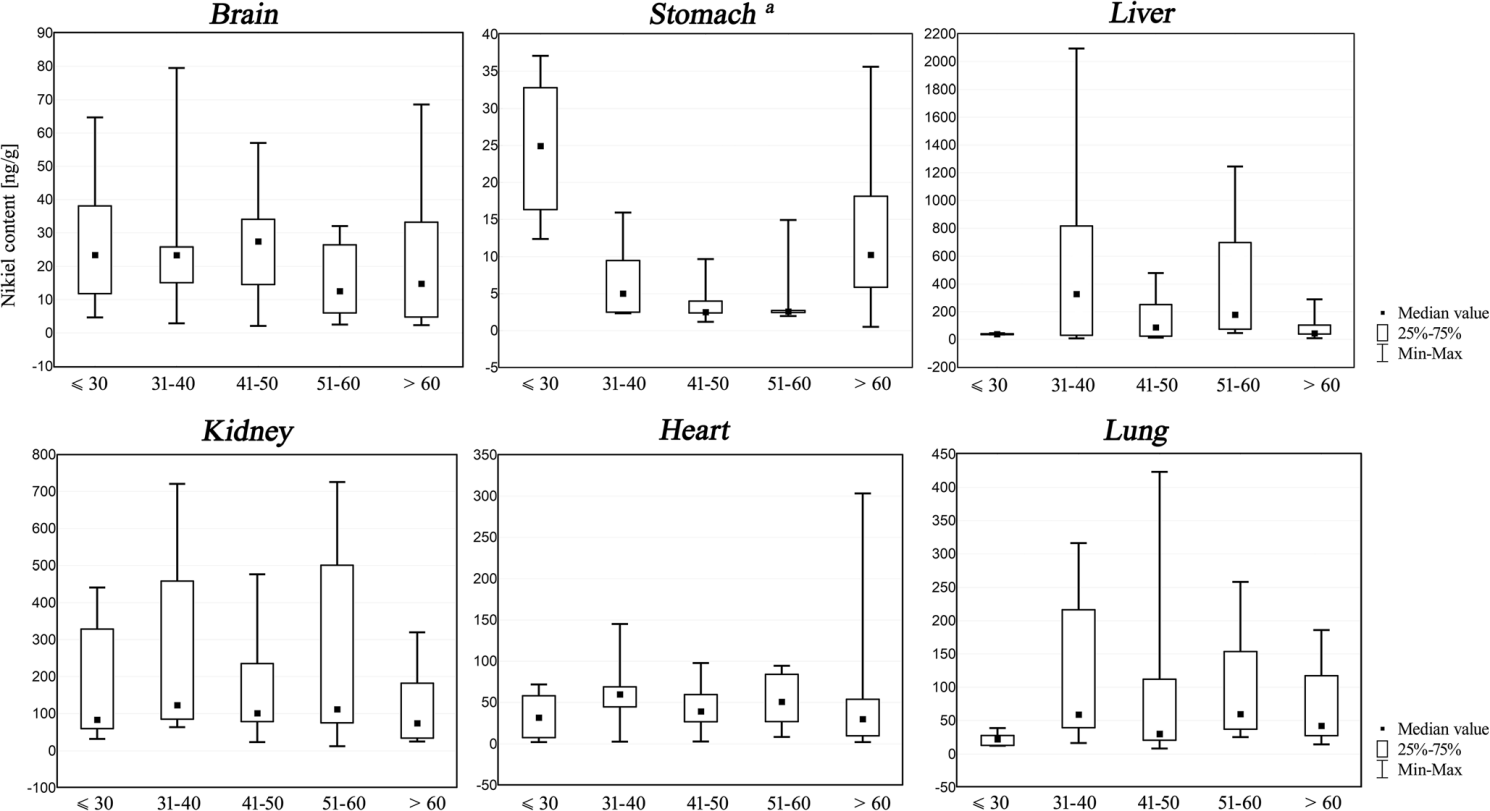
Nickel content in internal organs (ng/g) of the non-exposed population of Southern Poland: Kruskal-Wallis ANOVA test presented in box and whisker plots according to age-groups. **a** Statistically significant relationships between nickel content and age (*p* < 0.05) were revealed in stomach between age groups 1 and 2 (*p* = 0.03), 1 and 3 (*p* = 0.0002) and 1 and 4 (*p* = 0.007)

Statistica 5.0 software was applied to statistical analyses. Grubbs’ test for outliers was run to detect and reject outliers, if present, before statistical evaluation. The Mann-Whitney *U* and ANOVA Kruskal-Wallis ANOVA tests are chosen to assess the relationship between nickel content and gender or age of deceased―box and whisker plots are presented in Figs. 1 and 2.

## Results and Discussion

All values of nickel concentrations found in internal organs according to gender and age are presented in Tables 1 and 2, respectively. The tables summarize the mean, median and ranges of nickel concentration values of the analysed samples together with standard deviation values which can reflect the individual differences between subjects. Several extreme results detected in the analysed material (e.g. in determinations for total values: 97.5 ng/g in the brain; 423, 556 and 1951 ng/g in the lungs; 145, 220, 272 and 303 ng/g in the heart; 694, 803, 815, 869, 1215, 1245, 1771 and 2094 ng/g in the liver; and 59.2, 65.2, and 70.8 ng/g in the stomach) were excluded before statistical evaluation (Grubbs’ test).

**Table 1:** Nickel content in internal organs (number of samples, mean ± SD, median, range) in non-exposed population of Southern Poland (ng/g wet weight)―by gender

Material	Group	*n*^a^	Mean ± SD	Median	Range	
Brain	Female	20	18.1 ± 14.7	14.7	2.32	49.9
	Male	39	28.4 ± 21.2	24.4	2.12	79.4
	Total	59	24.9 ± 19.7	22.5	2.15	79.4
Stomach	Female	19	11.1 ± 10.1	9.68	0.5	35.6
	Male	34	9.28 ± 9.61	4.07	1.2	37.1
	Total	54	10.6 ± 10.7	7.5	0.5	44.2
Liver^b^	Female	17	44.0 ± 16.4	41.5	19.5	75.3
	Male	37	272 ± 335	138	7.85	1245
	Total	52	122 ± 131	49.5	7.85	519
Kidneys^b^	Female	19	74.7 ± 46.9	71.2	12.8	183
	Male	39	242 ± 216	131	38.1	725
	Total	60	196 ± 198	98.4	12.8	725
Lungs^b^	Female	16	25.0 ± 10.2	23.9	8.47	44.3
	Male	36	113 ± 114	58.3	11.3	423
	Total	54	83.4 ± 92.0	37.9	8.47	333
Heart	Female	19	37.2 ± 27.7	29.6	2.3	97.7
	Male	37	42.2 ± 26.4	40.4	2.3	94.5
	Total	56	40.5 ± 26.7	39.7	2.3	97.7

^a^Total number of samples―after discarding outlier values. ^b^Statistically significant relationships between nickel content and gender (*p* < 0.05) were revealed in the liver (*p* = 0.01), kidneys (*p* = 0.0006) and lungs (*p* = 0.001)

**Table 2:** Nickel content in internal organs (number of samples, mean ± SD, median, range) in non-exposed population of Southern Poland (ng/g wet weight)―by age group

Material	Age group	*n*^a^	Mean ± SD	Median	Range	
Brain	< 30	8	27.0 ± 19.5	23.5	4.67	64.7
	31–40	10	30.0 ± 26.6	23.4	2.88	79.4
	41–50	17	25.7 ± 15.4	27.4	2.12	57
	51–60	10	15.2 ± 10.8	12.6	2.51	32
	> 60	13	22.1 ± 19.9	14.8	2.32	68.6
Stomach^b^	< 30	7	24.0 ± 9.04	24.9	12.4	37.1
	31–40	10	6.44 ± 4.69	5.01	2.34	15.9
	41–50	13	3.69 ± 2.62	2.5	1.2	9.68
	51–60	9	5.20 ± 5.43	2.58	1.98	14.9
	> 60	12	12.5 ± 10.0	10.3	0.5	35.6
Liver	< 30	5	38.9 ± 4.1	37.7	35.2	45.2
	31–40	10	575 ± 680	328	7.85	2094
	41–50	18	142 ± 140	87.8	13.1	478
	51–60	11	370 ± 400	178	46	1245
	> 60	13	87.5 ± 89.5	43.9	8.48	289
Kidneys	< 30	8	177 ± 165	83.6	32.2	441
	31–40	10	263 ± 266	123	63.1	721
	41–50	18	166 ± 138	101	23.9	477
	51–60	11	246 ± 267	112	12.8	713
	> 60	12	119 ± 108	73.8	25.5	320
Lungs	< 30	7	22.3 ± 9.4	22.4	12.5	38.7
	31–40	9	111 ± 110	58.9	16.7	316
	41–50	16	95.7 ± 132	30.1	8.47	423
	51–60	9	102 ± 87.9	59.8	25.6	258
	> 60	12	73.6 ± 62.3	42.1	14.6	186
Heart	< 30	8	33.5 ± 27.9	31.5	2.3	71.8
	31–40	10	60.5 ± 37.0	59.8	2.7	145
	41–50	17	41.9 ± 25.1	39	3	97.7
	51–60	11	49.5 ± 31.0	50.8	8.47	94.5
	> 60	13	66.0 ± 100.7	29.6	2.3	303

^a^Total number of samples―after discarding outlier values. ^b^Statistically significant relationships (*p* < 0.05) were revealed between age groups 1 and 2 (*p* = 0.03), 1 and 3 (*p* = 0.0002) and 1 and 4 (*p* = 0.007)

In a previously published paper, the authors stated that the lungs have been found to contain the highest concentration of retained nickel in humans with no known occupational exposure, and the pulmonary burden of nickel has been shown to increase with age [4]. However, in the present study, such tendencies could not be seen, as the highest nickel content was determined in the liver, whilst the median values of nickel concentration in lungs, in all age groups, were practically on a similar level, which was lower than in kidneys and liver. Additionally, nickel concentrations in the stomach and brain were obviously lower than those in the liver and kidney. In particular organs, nickel concentrations had a wide range of values.

Figure 1 presents the effect of gender on the concentration of nickel in all considered organs. As can be seen and was tested using the Mann-Whitney *U* Test, females had generally lower nickel concentrations in the tissue samples than males. Statistically significant relationships between nickel content and gender (*p* < 0.05) were revealed in the liver (*p* = 0.01), kidneys (*p* = 0.0006) and lungs (*p* = 0.001).

Correlations between nickel content and age for five age groups are presented in Fig. 2. Median nickel concentrations in all studied material (with the exception of stomach) within all age groups had very similar values. The Kruskal-Wallis ANOVA test revealed statistically significant relationships in stomach―between age groups 1 and 2 (*p* = 0.03), 1 and 3 (*p* = 0.0002) and 1 and 4 (*p* = 0.007).

In order to compare our results with analytical data reported by other authors, wherever possible, country of origin, number of participants, age ranges, cause of death and values of mean, median and range of nickel in internal organs are collected from the literature and are listed in Tables 3 and 4. As can be seen, all papers deal with metal determination in fresh material, derived from both female and male subjects in a similar age range. There have been no data concerning nickel content in the stomach provided by other authors, and only two out of five papers reported nickel determination in four other organs [27, 28]. Caroli et al. [29] determined nickel concentration in the liver, kidneys and lungs; Drobyshev et al. [23] in the liver and kidneys; and Rahil-Khazen et al. [30] in the kidneys and heart. Most of the references are before 2016, because currently there are no new ones in which the problem of nickel determination and evaluation of reference values would appear. We mentioned that recently available literature on nickel content in human body mostly concerns the material which can be easily obtained from living subjects, such as blood, urine or hair. The availability of post-mortem material, organs in particular, is still limited for a large group of researchers.

**Table 3:** Comparison of populations investigated by various authors

Country	*n*	Age range	Material	Cause of death	Reference
Poland	39^a^	24–88	Fresh^d^	Not environmentally or occupationally exposed to elevated levels of nickel nor intoxicated with any poisons, and, on the basis of visual assessment of internal organs without any visible pathological or injury-related changes	This study
	21^b^	29–89			
	60^c^	24–89			
Russia	14^c^	Not stated	Fresh	Not stated	[23]
France	21^c^	19–57	Fresh	“Not professionally exposed to metals”	[27]
South Korea	89^a^	Not stated	Fresh	“Died prematurely, from trauma, traffic accident, hanging, strangulation, etc. (…) without special diseases”	[28]
	61^b^	Not stated			
	150^c^	12–87			
Italy	41^c^	Not stated	Fresh	“All individuals (…) had not been occupationally exposed to elements, had no known pathologies affecting any of the three organs”	[29]
Norway	13^a^	Not stated	Fresh^e^	“16–cases of sudden death, 14–inpatients who died from one or a combination of: cancer, heart failure, myocardial infarction, lung embolism, pneumonia, and septicaemia”	[30]
	17^b^	Not stated			
	30^c^	17–96			

^a^Male, ^b^female, ^c^total, ^d^less than 24 h from death to autopsy, and ^e^20–72 h from death to autopsy

**Table 4:** Nickel content in internal organs (number of samples, median, mean ± SD, range) found by various authors (ng/g)

Material	*n*	Mean ± SD	Median	Range		Determination technique	Reference
Brain	20	15		10	30	ICP-MS	[27]
	80^a^ 52^b^ 132^c^	70 ± 130 40 ± 70 60 ± 110		40 20 40	100^d^ 60^d^ 80^d^	ICP-AES	[28]
Liver	21	12		5.0	21	ICP-MS	[27]
	79^a^ 55^b^ 134^c^	90 ± 170 40 ± 70 70 ± 140		50 20 40	120^d^ 60^d^ 90^d^	ICP-AES	[28]
	41	150	100	10	550	ICP-AES	[29]
	14	180		40	600	AES	[23]
Kidneys	19	12		10	21	ICP-MS	[27]
	78^a^ 52^b^ 130^c^	100 ± 180 80 ± 100 90 ± 150		60 50 70	140^d^ 100^d^ 120^d^	ICP-AES	[28]
	41	90	60	10	70	ICP-AES	[29]
	14	140		30	600	AES	[23]
	21^e^ 21^f^		38 59	< LOD < LOD	189 225	ICP-AES	[30]
Lungs	20	16		10	52	ICP-MS	[27]
	72^a^ 56^b^ 128^c^	100 ± 150 130 ± 150 120 ± 150		70 90 90	140^d^ 170^d^ 140^d^	ICP-AES	[28]
	41	350	190	50	690^g^	ICP-AES	[29]
Heart	20	< LOQ				ICP-MS	[27]
	80^a^ 52^b^ 132^c^	70 ± 130 40 ± 700 60 ± 110		40 20 40	100^d^ 60^d^ 80^d^	ICP-AES	[28]
	18		54	< LOD	202	ICP-AES	[30]

^a^Male, ^b^female, ^c^total, ^d^95% confidence interval, ^e^kidney cortex, ^f^kidney medulla, and ^g^range as 5–95th percentile

The mean concentrations reported in this study, with the exception of the kidneys, were lower than those obtained by You et al. [28] for both men and women and, however, in all investigated matrices, higher than those published by Goullé et al. [27], who reported exceptionally low concentrations in all tissues. In comparison with studies from other parts of the world, nickel concentrations in the kidneys of subjects in Southern Poland reported in this study are about double the values reported in South Korea [28] and Italy [29] and five and even more than ten times greater than those obtained for the Norwegian [30] and French [27] population, respectively, yet similar to the levels reported from Russia [23].

The results obtained for nickel in liver samples in the range of 7.85–519 ng/g, with an average value of 122 ng/g, are in good agreement with those reported earlier by Drobyshev et al. [23] and Caroli et al. [29], in Russia and Italy, respectively. When comparing median values of nickel content in the lungs (37.9 ng/g) and heart (39.7 ng/g) with those reported by different authors mentioned in Table 4, it can be seen that they are lower than those found in Italian and Norwegian populations, respectively.

The differences between values for nickel determined during this study and those reported by other authors may partly be explained by different environmental exposure to this element in various countries, as well as different nutrition and various lifestyle factors. Additionally, the methodology of testing may also have an impact on the results obtained: for example, the method of sampling, storage and sample preparation as well as the analytical method. In all cited publications [23, 27–30], the authors used multi-elemental techniques that may be affected by various interferences to a greater or lesser extent.

## Conclusions

In conclusion, the results obtained for nickel content in human internal organs from 60 adults in the Polish non-environmentally and non-occupationally exposed population indicate that the data cover a wide range of concentrations―the lowest levels of nickel were found in the stomach and brain and the highest in the liver and kidneys in contrast to other authors who had mentioned the lungs. Values of nickel concentration in the lungs, regardless of gender, were on a similar level, which was about 50% lower in comparison with the levels in the kidneys and liver.

As the availability of post-mortem material, organs in particular, is still limited for a large group of researchers, the obtained data may constitute a contribution to population-based studies on metal content in biological material and be useful in the interpretation of the results of chemo-toxicological investigations.
